# Editorial: Student and teacher writing motivational beliefs

**DOI:** 10.3389/fpsyg.2024.1365757

**Published:** 2024-01-29

**Authors:** Steve Graham, Gustaf B. Skar, Gary A. Troia

**Affiliations:** ^1^Division of Leadership and Innovation, Mary Lou Fulton Teachers College, Arizona State University, Tempe, AZ, United States; ^2^Department of Teacher Education, Norwegian University of Science and Technology, Trondheim, Norway; ^3^Department of Counseling, Educational Psychology and Special Education, Michigan State University, East Lansing, MI, United States

**Keywords:** writing, motivation, teacher, student, beliefs

The study of writing has historically concentrated on what students write and how they write. This began to change in 1996 when John Hayes modified his seminal model of writing, arguing that motivation influences how we respond to immediate goals such as writing a particular paper for a given audience, but it also manifests into more long-term predispositions toward writing. While the study of motivational beliefs in writing preceded Hayes (1996) model (e.g., Graham and Harris, 1989), the inclusion of motivational beliefs in this model served as a catalyst for new investigations in this area (Camacho et al., 2021).

This increased and continuing interest in writing motivational beliefs is evident in recent reviews of the literature (Camacho et al., 2021) as well as theory (Graham, 2018). It is also evident in the current volume, which includes 17 chapters focused on writing motivational beliefs. More specifically, this volume brings together in a book collection studies examining the role of writing motivational beliefs across both sides of the desk: writer and teacher. The chapters and associated studies in this volume expand what we know about the motivational beliefs that drive (or inhibit) students' writing and that serve as catalysts for teachers' actions or inactions in the classroom.

## This volume

### Motivational beliefs and theory

The volume opens with a section on Motivation Beliefs and Theory, where Russell considered how concepts from genre, social action theory, and self-determination theory (Ryan and Deci, 2017) can expand the conceptualization of writing motivational beliefs. Russell also examined the possible implications of these viewpoints for research on student motivation, considering both sociocultural and cognitive perspectives.

### Measuring writing motivational beliefs

The second section of this volume begins with a chapter by DeBusk-Lane et al. that examined the multi-dimensionality of the popular Self-Efficacy for Writing Scale (SEWS). Through a series of measurement model comparisons, they validated that the SEWS is a multidimensional tool with a global theme and relevant sub-constructs: efficacy for conventions, self-regulation, and ideation. Using profile analyses, they also established three different patterns of writing self-efficacy among students (strongly inefficacious: conventions; moderately inefficacious; and efficacious: self-regulation).

Braten et al. designed and tested a new measure to assess students' efficacy for integrating information across multiple sources when writing. Using confirmatory factor analyses, they obtained evidence on the validity of the factor structure of the scale with undergraduate students. They also found that the scale was reliable and statistically associated with students' prior writing achievement, reading comprehension, and executive functioning.

Takada et al. conducted an exploratory mixed-methods study to determine how kindergarten children understand and respond to different methods of assessing motivational beliefs about writing (Likert-type survey, binary choice survey, a challenge preference task, and a semi-structured interview). They found that it was difficult to quantify the motivational beliefs of children this young. Additionally, kindergartners' views of motivation were multifaceted and contextually grounded.

### Students' motivational characteristics

The third section of the book focuses on students' motivational characteristics. While information on students' motivational characteristics is presented in other sections of the volume (see DeBusk-Lane et al. above), this section included two chapters that concentrate primarily on this topic. Cordero et al. used profile analysis to identify writing motivational and ability profiles for Grade 7 and 8 students participating in an automated writing evaluation intervention. They identified four distinct profiles and found that 30% of the students were likely to change their profile over the course of the school year. In the second study, Sehlström et al. examined if there were differences in the writing achievement and motivational beliefs of 8-year-old students with and without reading diffculties. Students who were better readers had higher writing efficacy and writing scores than weaker readers.

### Interplay between writing motivational beliefs and other aspects of writing

In the fourth section of this volume, three chapters examined the interplay between motivational variables and other aspects of writing (this also occurred to a lesser extent in other sections of the book, e.g., Braten et al.). Busse et al. assessed the interplay between writing efficacy, anxiety, and writing quality with students in Grade 9. They observed positive associations between writing efficacy and writing quality. Negative correlations were obtained between writing anxiety and writing quality. However, the associations between efficacy, anxiety, and writing quality were mediated by students' migration backgrounds.

Skar et al. also examined the interplay between writing efficacy and writing quality, but instead of determining how writing anxiety related to these variables, they focused on attitudes toward writing. They found that efficacy for writing self-regulation and attitudes toward writing each made unique contributions to predicting the quality of texts written by Grade 2 students. They further found that writing motivational beliefs were related to gender and language status (L1, bilingual, and L2).

In a third chapter, De Smedt et al. examined the relationship between writing self-efficacy and writing performance, but they extended their analyses to include measures of implicit theories of writing, writing motives, and achievement goals. Using path analysis, they found statistically significant direct paths between these writing motivational measures and the writing of 16- to 18-year-old students.

### Teachers' writing motivational beliefs

In the fifth section of this volume, three chapters concentrate on teachers' writing motivational beliefs. Wang and Troia provide the lead into this section by noting that students' motivation to write is not independent of the learning environment or teacher characteristics, including teachers' efficacy. Applying hierarchical linear modeling, they examined the relations among students' writing motivation, teacher efficacy for teaching writing and other professional traits, teachers' writing instruction, and the writing performance of Grade 4 and 5 students. While the analyses did reveal that the relationship between student motivation and achievement was moderated by writing instructional practices, teachers' efficacy was not uniquely related to how well students wrote.

The chapter by Bingham and Gerde focused just on early childhood teachers' writing beliefs and practices. They found that how teachers defined writing was unrelated to their beliefs about how children learn to write, but (1) teachers who defined writing as involving multiple writing skills were more likely to emphasize the relations between oral and written language in their instructional practices and draw attention to how English print works and (2) teachers' beliefs were positively associated with the number of spelling-related writing interactions they had with children.

In a study by Rouse et al., the instructional moves of preservice teachers during a simulated teaching situation involving writing conferences were observed. While the participants indicated that this simulation was useful and effective, teachers' efficacy for writing instruction was not clearly related to what preservice teachers did during the simulation.

### Writing motivational beliefs and instruction

The final section of this volume focuses on writing motivational beliefs and instruction. The first chapter by Wolbers et al. overlaps somewhat with the previous section on teachers' writing motivation beliefs. Teachers of students identified as deaf or hard of hearing were randomly assigned to a professional development (PD) treatment where they learned how to implement a strategy-oriented instructional approach to writing or a business-as-usual condition. The teachers implemented the writing practices taught during PD over the course of the school year. PD and subsequent implementation of the writing program enhanced the following teacher beliefs: writing interest, efficacy for teaching writing, and malleability of writing through effort and practice.

In the chapter by Seikmann et al., a pre-post quasi-experiment was conducted with Grade 9 German students learning English as a foreign language. The students were provided feedback on their writing for an eight-month period. From the start of the school year to the end of it, students' perceptions of the quality of the feedback improved as did their writing self-efficacy, whereas writing anxiety decreased.

Fulton et al. conducted a quasi-experiment with high school students. Their study compared the impact of a dialogic literary argumentation program to a close reading program. Both of these programs improved the argumentative writing of participating students, with the dialogic group making the most growth. While neither of the groups evidenced changes in writing motivational beliefs, the writing motivational beliefs of students in the dialogic group were more positively correlated with their writing performance at posttest than for the close reading group.

Myhill et al. investigated how students aged 7 to 14 years responded to a changed classroom environment for writing. They found that such a change had a positive impact on students. Specifically, they enjoyed more autonomy and choice by the end of the writing treatment and experienced their writing classrooms as more relaxed.

In the final chapter in this volume, Collins et al. assessed how the writing motivations of international students attending university in the United States changed as they completed an online academic course. They found some evidence that participating students' writing motivations were malleable, as increased levels of student writing self-efficacy were evident by the end of the course. While writing self-efficacy at the start of the course positively predicted writing performance, students' beliefs about writing as a tool for exploring and expressing ideas was associated with lower odds of passing the course.

## Concluding comment

In closing, we hope you enjoy reading the studies presented in the chapters in this volume as much as we did. We also hope they stimulate you to think about teacher and student writing motivational beliefs more broadly and more creatively.

## Author contributions

SG: Writing – original draft. GS: Writing – review & editing. GT: Writing – review & editing.
